# New Binding Mode to TNF-Alpha Revealed by Ubiquitin-Based Artificial Binding Protein

**DOI:** 10.1371/journal.pone.0031298

**Published:** 2012-02-20

**Authors:** Andreas Hoffmann, Michael Kovermann, Hauke Lilie, Markus Fiedler, Jochen Balbach, Rainer Rudolph, Sven Pfeifer

**Affiliations:** 1 Institute of Biochemistry and Biotechnology, Martin Luther University Halle-Wittenberg, Halle, Germany; 2 Institute of Physics, Martin Luther University Halle-Wittenberg, Halle, Germany; 3 Scil Proteins GmbH, Halle, Germany; National Institute for Medical Research, Medical Research Council, London, United Kingdom

## Abstract

A variety of approaches have been employed to generate binding proteins from non-antibody scaffolds. Utilizing a beta-sheet of the human ubiquitin for paratope creation we obtained binding proteins against tumor necrosis factor (TNF)-alpha. The bioactive form of this validated pharmacological target protein is a non-covalently linked homo-trimer. This structural feature leads to the observation of a certain heterogeneity concerning the binding mode of TNF-alpha binding molecules, for instance in terms of monomer/trimer specificity. We analyzed a ubiquitin-based TNF-alpha binder, selected by ribosome display, with a particular focus on its mode of interaction. Using enzyme-linked immunosorbent assays, specific binding to TNF-alpha with nanomolar affinity was observed. In isothermal titration calorimetry we obtained comparable results regarding the affinity and detected an exothermic reaction with one ubiquitin-derived binding molecule binding one TNF-alpha trimer. Using NMR spectroscopy and other analytical methods the 1∶3 stoichiometry could be confirmed. Detailed binding analysis showed that the interaction is affected by the detergent Tween-20. Previously, this phenomenon was reported only for one other type of alternative scaffold-derived binding proteins – designed ankyrin repeat proteins – without further investigation. As demonstrated by size exclusion chromatography and NMR spectroscopy, the presence of the detergent increases the association rate significantly. Since the special architecture of TNF-alpha is known to be modulated by detergents, the access to the recognized epitope is indicated to be restricted by conformational transitions within the target protein. Our results suggest that the ubiquitin-derived binding protein targets a new epitope on TNF-alpha, which differs from the epitopes recognized by TNF-alpha neutralizing antibodies.

## Introduction

The use of antibodies dominated the development of molecules with specific binding properties in the past decades. However, they show particular limitations arising mainly from their architecture. The composition of full-size antibodies from four polypeptide chains (two heavy and two light chains) that are connected by inter-chain disulfide bonds demands complicated cloning steps and a production using secretory pathways. The large molecular size of complete antibodies limits penetration of dense tissue, reducing applicability in e.g. solid tumor therapy. By using the F_v_ fragment, containing the whole antigen binding site of an antibody, the molecule size can be reduced. However, reduced stability of F_v_ fragments requires the employment of protein engineering to obtain suitable molecules for biotechnological and medical applications [1].

As a complement to antibodies, alternative scaffold proteins were utilized to develop molecules with the desired properties regarding binding, architecture and stability [2]–[6]. Almost all of these scaffolds consist of a single polypeptide chain and lack intrinsic cysteines, offering the possibility for intracellular expression in bacteria. A high thermodynamic stability gives the opportunity of creating a binding surface by extensive substitution of amino acids without disturbing the overall fold of the protein or its solubility. Furthermore, most of the scaffold proteins are chosen to be small protein molecules with a molecular weight of less than 15 kDa. Different alternative scaffolds were successfully used to create artificial binding proteins against a number of target molecules from various classes. Affinities comparable to antibodies, down to the picomolar range, were obtained e.g. for tetranectins, anticalins, DARPins and affibodies [4], [7]–[9]. The diversity of the scaffolds almost coincides with the number of approaches to create a target specific binding area in terms of localization and method of amino acid substitution. Often, but not necessarily, amino acid positions known to be involved in natural interactions of the scaffold are used as a starting point for the generation of tailored binding properties, for example in the lipocalin-derived anticalins and the affibodies [5], [7]. In analogy to technologies for the *in vitro* generation of recombinant antibodies, engineering of these potential paratopes by creating diverse libraries with subsequent selection by evolutionary methods yields target-binding protein variants.

In the context of the particular scaffold, the selection of the amino acid positions to be involved in paratope formation offers the possibility to influence the epitope selectivity and the thermodynamic parameters. By employing particular secondary structures for creation of the paratope, its rigidity and thus the entropic effects on binding can be affected. The loops that are mediating the binding properties in antibodies and several scaffolds often show structural rearrangements upon target interaction. Fixation of mobile loops during the binding event results in unfavorable entropic contributions. A higher initial complementarity of the paratope with the epitope geometry, together with a diminished intrinsic flexibility, permits reduction of entropic losses, though on the risk of reducing variability in target binding. Indeed, examples of antibodies after affinity maturation show a decrease in entropic costs for complex association, resulting from structural pre-organization of the binding site [10]. Consequently, harnessing rigid secondary structures of an alternative scaffold should in principle reduce negative entropic effects.

Such an approach to generate a universal binding site upon rigid secondary structures uses the helices of staphylococcal protein A for paratope creation. Furthermore, the beta-sheet structures of human gammaB-crystallin and human ubiquitin were employed to create *de novo* binding sites [11], [12]. In contrast to the *de novo* generation of such a paratope, in this work the knowledge on the well-characterized binding site in the ubiquitin for the ubiquitin-binding domains (UBD) should be used for creation of a new ubiquitin variant library. This binding area is located at a hydrophobic patch comprising L8, I44 and V70 [13], [14]. The dissociation constants, reported for most of the UBDs, range from micro- to millimolar values [15], [16]. Using these amino acid positions as a starting point for the paratope generation, we aimed to create ubiquitin-based binders with higher affinities, at least in the nanomolar range.

For validation of the feasibility of this strategy to generate binders from the scaffold ubiquitin, the tumor necrosis factor (TNF)-alpha was chosen as a target protein. The cytokine TNF-alpha was originally discovered by its activity of killing tumor cells *in vitro*. Today it is a well established pharmacological target for the treatment of inflammatory diseases, e.g. rheumatoid arthritis and Crohn's disease. Currently, etanercept, the TNF-receptor 2 dimerized by fusion to the IgG1 F_c_ fragment, and two monoclonal antibodies, i.e. infliximab and adalimumab, are approved TNF-inhibiting biologicals [17]. Besides efforts to create TNF-inhibiting antibodies, various attempts of selection with alternative scaffolds were made against this protein. To compete with the TNF-receptor for binding to TNF-alpha, the biologically active TNF-inhibitors need extraordinary high affinities. Typically, dissociation constants in the subnanomolar range are required [4], [17]. The homotrimeric structure of the cytokine leads to the observation of certain heterogeneity concerning the oligomer/monomer-specificity of TNF-alpha binders. For example, etanercept recognizes only the homotrimer form of TNF-alpha. It binds to an epitope which involves two subunits simultaneously [18]. In contrast, most TNF-alpha neutralizing monoclonal antibodies, binding only one subunit, recognize also the monomeric form [17], [19], [20].

In the present study we show a detailed molecular characterization of an artificial binding protein, selected *in vitro* from a ubiquitin F45W-derived library against TNF-alpha. To examine the ubiquitin variant-TNF-alpha interaction, including the effect of the detergent Tween-20, several methods such as ELISA, isothermal titration calorimetry and NMR spectroscopy were utilized. Additionally, biophysical parameters of the selected binder were evaluated by spectroscopic methods. We find that the ubiquitin variant binds specifically the trimer form of the target molecule TNF-alpha with submicromolar affinity. The epitope accessibility can be enhanced by the detergent Tween-20, an observation according to our knowledge never reported for TNF-alpha binding antibodies before.

## Materials and Methods

### Materials

All chemicals and culture media were obtained from Roth (Karlsruhe, Germany), Merck (Darmstadt, Germany) and Applichem (Darmstadt, Germany) unless stated otherwise. DNA modifying enzymes were obtained from Fermentas (Roth, Germany). Chromatography materials and devices were from GE Healthcare (Uppsala, Sweden).

### Production, purification and chemical modification of TNF-alpha

The target protein TNF-alpha was produced by SUMO-fusion strategy as published previously [21]. The product identity was confirmed by mass spectrometry. An L929 cell assay was performed to compare the biological activity with commercially available TNF-alpha.

For chemical labeling of the TNF-alpha, Sulfo-NHS-LC-biotin (Pierce Biotechnology, Rockford, IL) was used. To obtain preferentially N-terminally labeled proteins reactions were performed at pH 6.5. Further reaction conditions were kept according to manufacturer's instructions. Successful modification was confirmed by mass spectrometry.

To avoid confusion the concentration of TNF-alpha is given per monomer for all experiments in this manuscript.

### Library construction, selection and binding variant identification

As scaffold basis a ubiquitin variant with an F45W substitution was used to allow routine spectroscopic characterization without relinquishing the positive properties of ubiquitin, particularly in terms of stability [22]. For the ubiquitin library generation, six amino acid positions of the scaffold were selected for randomization based on an analysis of involvement in natural interactions of ubiquitin [13], *in silico* algorithms calculating the stability effects of amino acid substitutions [12], and visual inspection using the open source software PyMOL version 0.99rc6 (DeLano Scientific LLC, South San Francisco, CA). Subsequently, overlapping oligonucleotides containing NNK motifs at the randomized positions were used for PCR-based library construction. With the resulting DNA library six selection cycles of ribosome display were performed [23], [24]. The isolated cDNA from the last selection cycle was subcloned into a modified pET-20b vector. After transformation of the ligation product into *Escherichia coli (E. coli)* NovaBlue(DE3), 192 clones were cultivated for 20 h at 37°C in 96-deep well format using 400 µl auto-induction medium ZYM-5052 [25]. The cells were harvested by centrifugation for 10 min at 4000× g. Subsequently, the pellets were resuspended in PBS (137 mM NaCl, 8 mM Na_2_HPO_4_, 2.7 mM KCl, 1.5 mM KH_2_PO_4_ pH 7.4) supplemented with 200 µg ml^−1^ lysozyme and BugBuster (Novagen, Darmstadt, Germany) and incubated for 10 min at 25°C. The lysate was cleared by centrifugation for 30 min at 5000× g and the supernatant was diluted ten-fold for use in ELISA. Binding of the ubiquitin variants to the target TNF-alpha and to lysozyme as a control protein was detected by anti-Flag-antibody M2-peroxidase conjugate (Sigma-Aldrich, St. Louis, MO). The target-binding variant with the highest binding signal, termed 10F, was chosen for further characterization. For this purpose, the genes of human ubiquitin F45W (optimized for expression in *E. coli*; GENEART, Regensburg, Germany) and the variant 10F (without Flag-tag) were subcloned into a modified vector pET-20b. The coding sequence of 10F_DY_ was obtained by inserting the codons for the amino acids D58 and Y59 into the 10F gene by standard-PCR methods and was subcloned into pET-41b (Novagen, Darmstadt, Germany).

### Production and purification of ubiquitin variants

For purification, ubiquitin F45W and its variants were produced in *E. coli* BL21(DE3) using auto-induction media ZYM-5052 for unlabeled proteins or N-5052 containing ^15^NH_4_Cl (Sigma-Aldrich, St. Louis, MO) for ^15^N-labeled proteins, respectively [25]. Following incubation for 20 h at 37°C and 220 rpm, cells were harvested, resuspended in NPI-20 (20 mM Na_2_HPO_4_, 150 mM NaCl, 20 mM imidazole pH 8.0) and disrupted as described previously [21]. The lysate was cleared by centrifugation and the supernatant was applied on an ÄktaXpress system to a 5-ml HisTrap HP column equilibrated with buffer NPI-20. After a washing step comprising 7 column volumes of NPI-40 (NPI-20, but 40 mM imidazole), the target protein was obtained by elution using NPI-500 (NPI-20, but 500 mM imidazole). Protein-containing fractions from the elution step were loaded on a HiLoad 16/60 Superdex 75 pg column for size exclusion chromatography using PBS, including 1 mM EDTA pH 7.4, as running buffer at a flow-rate of 1.0 ml min^−1^.

### ELISA

The immobilization was performed by overnight incubation at 4°C with 50 µl well^−1^ antigen in PBS on Maxisorp plates (Nunc, Wiesbaden, Germany). Then wells were blocked with 300 µl PBS, 1% (w/v) BSA for 2 h at 37°C. Each of the following incubation steps was preceded by three times washing with 300 µl PBS supplemented with 0.05% (v/v) Tween-20 (PBST0.05). His_6_-tagged or biotinylated proteins were allowed to bind to immobilized target for up to 2 h. For competition ELISA, the proteins were incubated with competitor at least 1 h before (on ice) and during the binding reaction. Bound protein was detected by anti-His_6_-mAB-peroxidase conjugate (Roche Applied Science, Mannheim, Germany) or avidin-peroxidase conjugate (Pierce Biotechnology, Rockford, IL) diluted in PBST0.05 using TMB Plus (Kem-En-Tec Diagnostics, Taastrup, Denmark) as substrate.

### Isothermal titration calorimetry

TNF-alpha and the ubiquitin variant 10F were dialyzed exhaustively against PBS, 0.5 mM EDTA pH 7.4 and subsequently supplemented with Tween-20 to a final concentration of 0.05% (v/v). Isothermal titration calorimetry was performed using a MicroCal VP-ITC titration microcalorimeter (MicroCal Inc., Northampton, MA) at 25°C. A series of 10-µl injections of 10F (135 µM) were added sequentially into TNF-alpha (35.6 µM). The heats of dilution for injecting 10F into measurement buffer were subtracted from binding experiments before curve fitting.

### Analytical size exclusion chromatography

TNF-alpha and the ubiquitin variant 10F were prepared in running buffer (PBS, 1 mM EDTA pH 7.4, supplemented with 0.05% (v/v) Tween-20 if necessary) to analyze the complex formation. Aliquots were loaded on a Superose 12 10/300 GL column at a flow-rate of 0.6 ml min^−1^.

### NMR spectroscopy

All NMR experiments were carried out on a Bruker 600 Avance III spectrometer equipped with a 5 mm triple inverse probehead for TNF-alpha titration experiments to 10F and on a Bruker 800 Avance III spectrometer equipped with a 5 mm triple inverse cryoprobe for recording free state ^1^H-^15^N fHSQC spectra [26] of ubiquitin F45W, 10F and 10F_DY_. Spectra were measured in PBS, 1 mM EDTA pH 7.4, containing 10% (v/v) D_2_O at 25°C.

The NMR titration of TNF-alpha to the ^15^N-labeled ubiquitin variant 10F was performed under equilibrium conditions in the presence and in the absence of 0.05% (v/v) Tween-20. For every step during the titration we followed the peak intensity and the chemical shift value of every 10F resonance in a series of ^1^H-^15^N fHSQC spectra of the ^15^N-labeled 10F. All NMR spectra were processed using NMRPipe [27] and analyzed using NMRView [28]. We ended up with a 12-fold excess for both titration experiments of TNF-alpha to 10F (1890 µM TNF-alpha, 160 µM 10F). As the particular chemical shift value for every cross peak representing the free state of 10F did not change during the whole titration experiment but the particular peak intensity decreases, the slow NMR chemical exchange limit between free and TNF-alpha-bound state of 10F was assumed for data analysis. Every amide proton intensity was dilution corrected for quantitative data analysis.

### Far-UV circular dichroism spectroscopy

To analyze the secondary structure of the ubiquitin variants, far-UV circular dichroism (CD) experiments were performed on a Jasco J-810 spectropolarimeter (Jasco Inc., Easton, MD, USA) at 25°C in a 1 mm quartz cuvette for native samples or 0.1 mm for guanidine hydrochloride denatured samples. Measurements ranged from 185 to 260 nm at a scanning rate of 10 nm min^−1^ and a bandwidth of 1 nm. All spectra were accumulated 20 times with a response time of 4 s. For recording spectra of the natively folded proteins concentrations of 7.01 µM for 10F, 10.16 µM for ubiquitin F45W, and 8.25 µM for 10F_DY_ in 10 mM KH_2_PO_4_ pH 7.4, respectively, were used. Measurements for protein samples denatured in 6 M guanidine hydrochloride were performed with 62.0 µM 10F and 32.5 µM ubiquitin F45W. The buffer-corrected spectra were converted to mean residue ellipticity according to Schmid [29].

Further methods are described in Methods S1.

## Results

### Selection and identification of TNF-alpha binding ubiquitin variants

**Figure 1:**
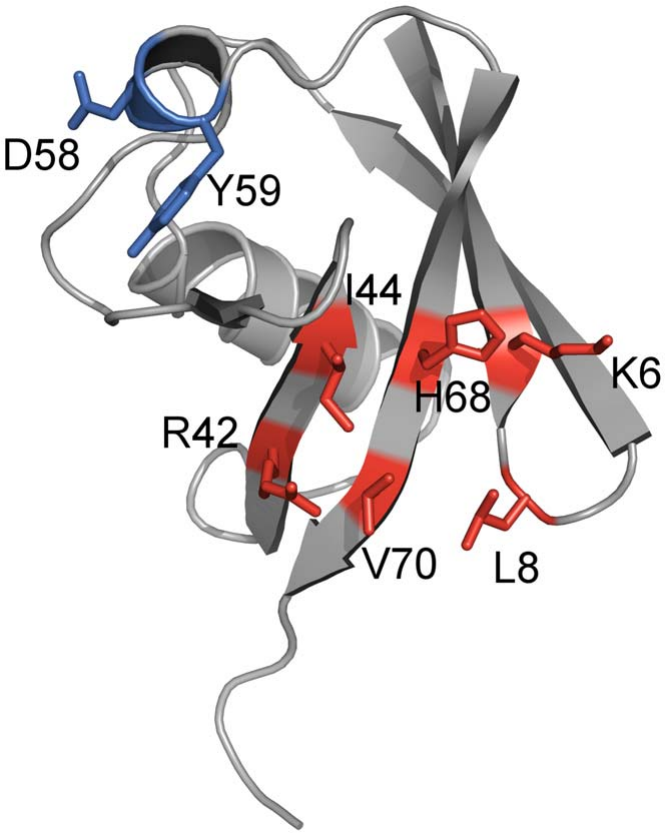
Amino acid positions chosen for randomization in library construction. Based on the scaffold ubiquitin (shown here in cartoon representation), in particular with an F45W substitution, a library for the selection of artificial binding proteins was generated. For this purpose the six surface-exposed amino acid residues K6, L8, R42, I44, H68 and V70 (highlighted in red), located in the beta-sheet region of the scaffold, were chosen to be randomized for library construction. After *in vitro* selection against TNF-alpha, in the ubiquitin variant named 10F the residues D58 and Y59 (highlighted in blue) were found deleted. This figure was generated using pdb entry 1UBI and the software PyMOL version 0.99rc6 (DeLano Scientific LLC, South San Francisco, CA).

Several considerations influenced the construction of the ubiquitin F45W-based library. First, as the basis for a paratope with new binding properties amino acid residues in the beta-sheet involved in natural interactions with ubiquitin-binding proteins were identified by thorough literature analysis on ubiquitin complexes as well as functional sequence alignments of ubiquitin-like proteins. Furthermore, tolerance of selected positions against random substitution was deduced by *in silico* algorithms [12]. Finally, by additional visual inspection of the 3D-model of ubiquitin (pdb-code 1UBI) using PyMOL software the six amino acid residues K6, L8, R42, I44, H68 and V70 were chosen for randomization (Fig. 1). These residues are located in the beta-sheet and form a contiguous surface area of approximately 475 Å^2^.

Based upon this residue selection, the DNA library was created by PCR-based gene synthesis with degenerated oligodeoxynucleotides to introduce randomized codons at the six selected positions. The TNF-alpha binding ubiquitin variant characterized in this work was isolated from this library using ribosome display selection. Although the naïve library comprised only six randomized amino acid positions (Fig. 1) the variant analyzed here in detail showed altogether nine modifications. In addition to the six expected substitutions at the randomized amino acid positions (K6F, L8Y, R42A, I44E, H68P, V70F), the exchange K29R, located in helix 1 of ubiquitin, as well as the deletion of D58 and Y59 in the loop, leading into the last beta-strand, were found. Since the deletions were assumed to have a major impact on structure and stability of the ubiquitin variant the two amino acids were re-inserted resulting in the variant 10F_DY_. For further experiments 10F, 10F_DY_ and ubiquitin F45W were produced as His_6_-tagged proteins in *Escherichia coli* BL21(DE3) and purified via IMAC and a subsequent size-exclusion chromatography step.

### Binding characterization by ELISA methods

**Figure 2:**
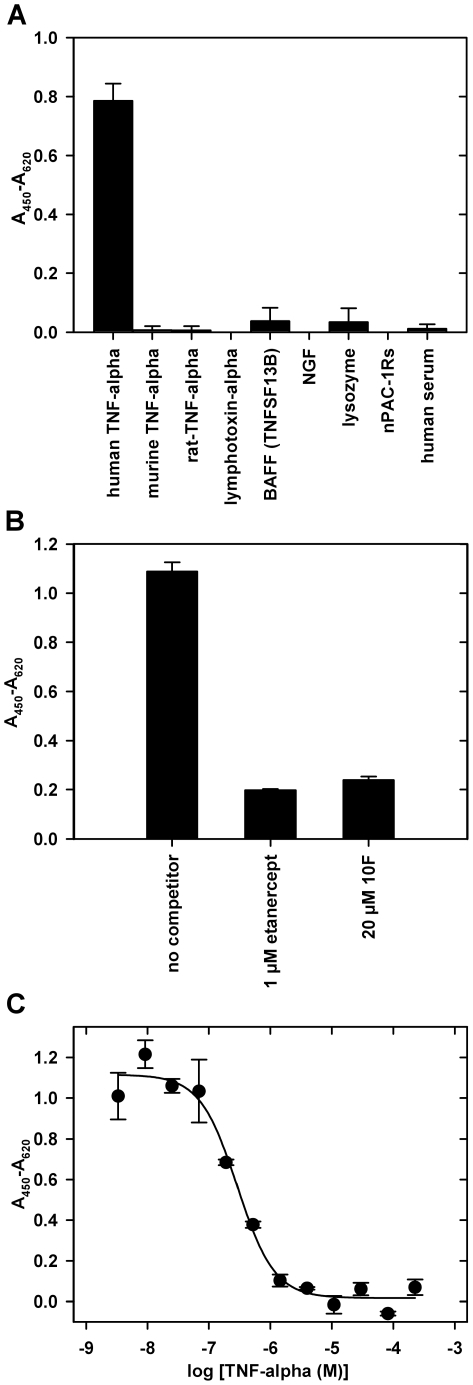
ELISA analysis of the TNF-alpha binding ubiquitin variant 10F. (A) Specificity of ubiquitin variant 10F. The interaction of the purified, His_6_-tagged binder with different immobilized target proteins (500 ng per well, human serum was used without dilution) is shown. (NGF: nerve growth factor, nPAC-1Rs: short splice form of N-terminal domain of human PACAP-receptor 1) (B) Characterization of the specificity by competition of the interaction of TNF-alpha and 10F. Biotinylated TNF-alpha (1 µM), preincubated for 1 h with competitor (etanercept: TNF-receptor 2 dimerized by fusion to IgG F_c_), was incubated with immobilized 10F (100 ng per well). Detection by avidin-peroxidase demonstrates competition by etanercept. (C) Concentration-dependent competition ELISA. 10F (100 nM, His_6_-tagged), preincubated with different concentrations of TNF-alpha, was incubated with immobilized TNF-alpha (150 ng per well). An IC_50_ value of 289±53 nM was determined.

All binding studies were performed in the presence of 0.05% (v/v) Tween-20 because the detergent was present in the primary selection as well. In ELISA studies only the ubiquitin variant 10F was able to bind, but not 10F_DY_ nor ubiquitin F45W (Fig. S1). Furthermore, ELISA experiments confirmed that 10F binds specifically to human TNF-alpha and shows no interaction with other proteins sharing up to 80% sequence similarity (Fig. 2A).

In a competition ELISA we were able to inhibit the binding of biotinylated TNF-alpha to immobilized variant 10F with etanercept (TNF receptor-IgG1-F_c_-part fusion) and with the variant 10F itself (Fig. 2B). A similar ELISA setup was used to obtain information on the affinity of the ubiquitin variant. Binding of 10F to immobilized TNF-alpha was competed by different concentrations of soluble TNF-alpha resulting in an IC_50_-value of 0.29±0.05 µM (Fig. 2C).

### Characterization of complex formation and stoichiometry

**Figure 3:**
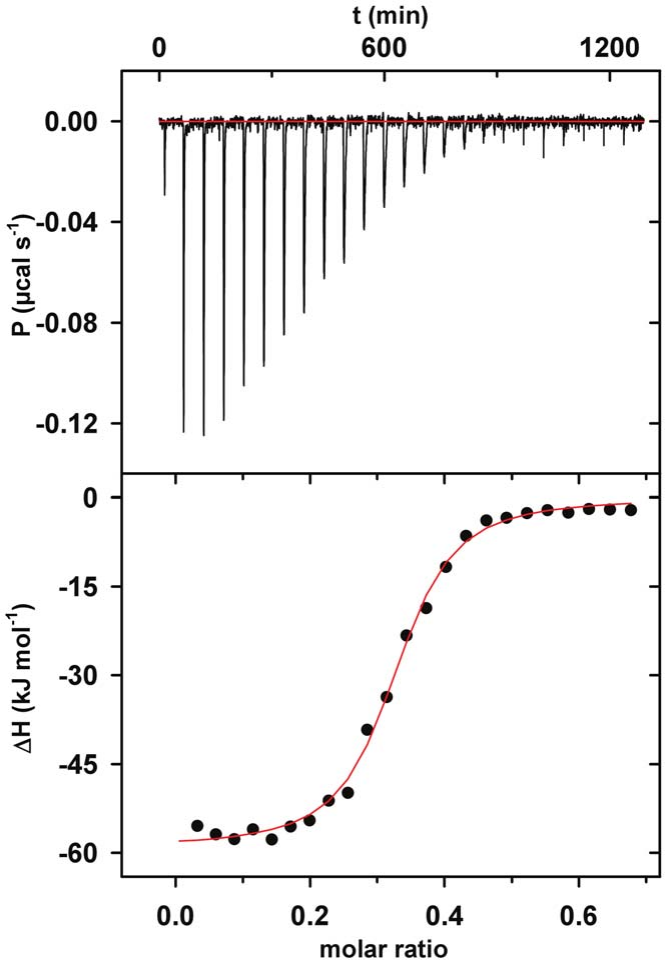
Isothermal titration calorimetry of TNF-alpha and the ubiquitin variant 10F. 10F at 135 µM was injected into the measurement cell containing TNF-alpha at 35.6 µM. The titration was carried out at 25°C. Experiments were conducted in duplicate. The upper panel shows the raw data and the lower panel shows the integrated enthalpy data corrected for dilution heat of a single experiment. Fitting the data to a single-site binding model (solid line), we determined K_D_ = 180±50 nM, stoichiometry factor N = 0.35±0.04, ΔH = −57.3±2.5 kJ mol^−1^ and TΔS = −18.8±3.2 kJ mol^−1^.

To characterize the thermodynamics of the interaction of 10F and TNF-alpha isothermal titration calorimetry experiments were performed. Comparable to the IC_50_-value obtained in ELISA, a K_D_ value of 0.18±0.05 µM was observed (Fig. 3). The 10F molecule was bound in an exothermic, enthalpy driven reaction (ΔH = −57.3±2.5 kJ mol^−1^; TΔS = −18.8±3.2 kJ mol^−1^) with one 10F molecule binding to one TNF-alpha trimer (N = 0.35±0.04).

**Figure 4:**
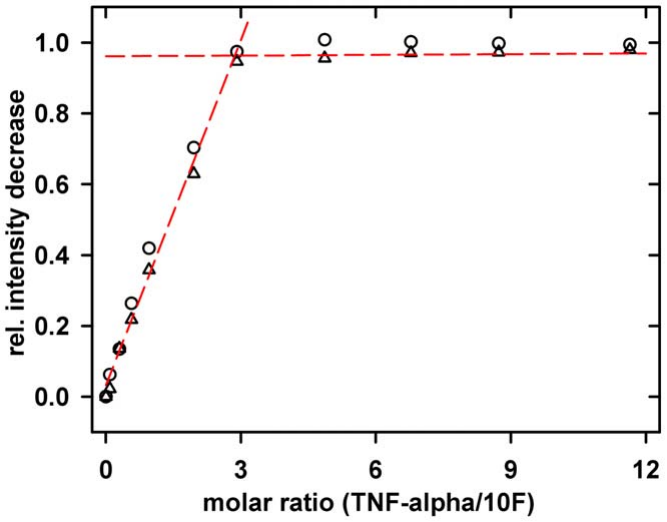
NMR analysis of the titration of 10F with TNF-alpha. ^1^H-^15^N fHSQC spectra of ^15^N-labeled ubiquitin variant 10F in presence of increasing concentration of unlabeled TNF-alpha were recorded in PBS, 1 mM EDTA pH 7.4 with 0.05% Tween-20. The relative intensity decrease of two amid resonances was plotted against molar ratio of both proteins. As determined by the intersection point of the linear fits of the initial part and the plateau part of the curve 2.88±0.18 TNF-alpha monomers are bound per 10F molecule.

Binding analysis between TNF-alpha and the ubiquitin variant 10F was followed on a residue-by-residue basis using NMR spectroscopy. A series of ^1^H-^15^N fHSQC spectra of the 10F variant with increasing amounts of TNF-alpha was recorded (in presence of 0.05% (v/v) Tween-20). Almost all chemical shift positions seen for the free 10F had changed to new values in the complex state (Fig. S2A). Acquisition of one 2D correlation spectrum of ^15^N-labeled 10F immediately after each addition of TNF-alpha, showed a linear decrease of essentially each individual cross peak representing the free state of the 10F variant until a stoichiometry of three TNF-alpha subunits per 10F monomer (Fig. 4). The repetition of the titration experiment in absence of Tween-20 led neither to shifting of any amide resonance nor to additional NMR signals of the TNF-alpha-bound state of 10F, even until the final titration step. This suggested that during the individual NMR titration steps the binding equilibrium had not been reached. Surprisingly, an incubation time for the final complex solution of 12 h leads to a complete population of the TNF-alpha-bound state of the 10F variant. Both ^1^H-^15^N spectra representing the complex state (the 12 h-incubated complex in absence of detergent and the 0.05% (v/v) Tween-20 containing final state) are fully superimposable (data not shown).

The complex stoichiometry was validated by equilibrium sedimentation (analytical ultracentrifugation) of fluorescein-labeled 10F (Fig. S3). Using absorbance measurement at 490 nm allowing specific detection of the fluorescein labeled ubiquitin variant, a molecular mass of 11±1 kDa was found for 10F (calculated mass 10 kDa) in absence of TNF-alpha. Upon addition of an excess of TNF-alpha the molecular mass shifted to 65±5 kDa corresponding to the calculated molecular mass of the TNF-alpha trimer-10F complex (62 kDa). A fluorescence homoquenching assay [30] was used to follow the monomer exchange between TNF-alpha trimers in presence and absence of TNF-alpha binding molecules (Fig. S4). Complex formation of TNF-alpha with binding partners recognizing the trimeric species by binding several subunits simultaneously should have a stabilizing effect, thus decreasing the monomer exchange rate. Indeed, upon addition of etanercept, known to bind the trimer form of TNF-alpha, an almost complete inhibition of the exchange was observed. The ubiquitin variant 10F decreased the exchange rate significantly as well, but not completely. In contrast, after addition of the variant 10F_DY_, showing no affinity for TNF-alpha at all, the rate of fluorescence increase was unaltered compared to the buffer control.

**Figure 5:**
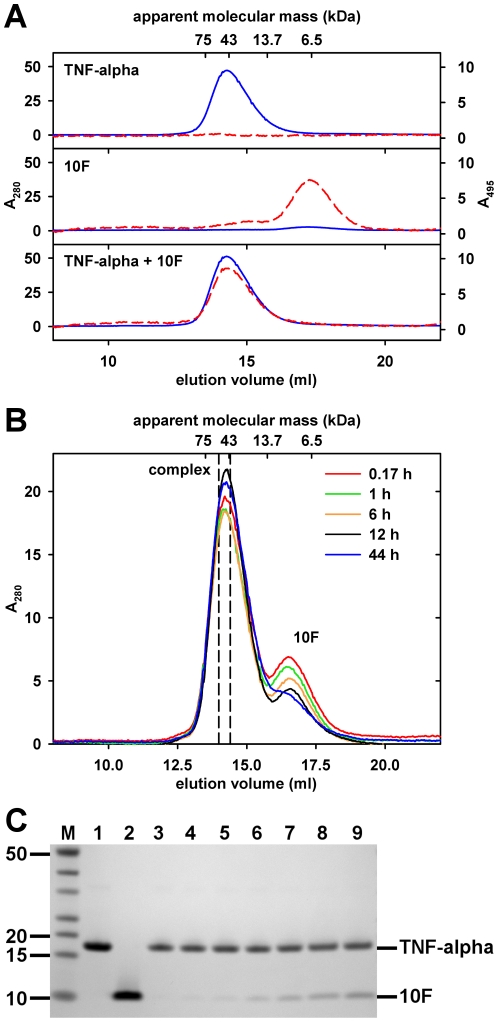
Size exclusion chromatography (SEC) of the complex of TNF-alpha and the ubiquitin variant 10F. (A) SEC in presence of detergent Tween-20. Samples of fluorescein-labeled 10F (10 µM), TNF-alpha (60 µM), and a mixture of both, were pre-incubated for 10 min at 4°C and loaded on a Superose 12 size-exclusion column. Experiments were performed with sample and running buffer, both containing 0.05% (v/v) Tween-20, and detection wavelengths of 280 nm (blue line) and 495 nm (red dashed line). Note that there is no significant shift of the TNF-alpha elution volume when comparing the elution profiles of TNF-alpha alone and the mixture. Fluorescein itself did not co-elute with TNF-alpha (data not shown). (B) SEC in absence of detergent Tween-20. For analysis of Tween-20 independent complex formation, a detergent-free mixture of unlabeled 10F (10 µM) and TNF-alpha (60 µM) was incubated at 4°C and aliquots were analyzed as described before using running buffer without Tween-20. Note that the area of the 10F peak (16.5 ml) decreases during prolonged incubation. Dashed lines mark the borders of the fraction used for SDS-PAGE analysis. (C) SDS-PAGE analysis of the detergent-free SEC complex fractions. Aliquots of appropriate SEC fractions were analyzed by SDS-PAGE (4–12% gradient gel) followed by Coomassie staining. M: Fermentas PageRuler Unstained, 1: control TNF-alpha (2 µg), 2: control 10F (2 µg), 3: 0.17 h preincubation, 4: 1 h, 5: 2 h*, 6: 6 h, 7: 12 h, 8: 24 h*, 9: 44 h. *To reduce complexity chromatograms were not included in (B).

To verify the results on the Tween-20 dependence of the interaction observed in the NMR experiments, size-exclusion chromatography experiments were conducted. To show complex formation, the target protein TNF-alpha, the fluorescein-labeled ubiquitin variant 10F or a mixture of both, respectively, were loaded onto an analytical gel filtration column. In presence of the detergent Tween-20, 10 min of incubation were sufficient for complete formation of the complex (Fig. 5A). By analogy with the NMR measurements, in the absence of Tween-20 binding of unlabeled 10F by TNF-alpha occurred only in the time-range of hours (Fig. 5B). Corresponding to the peak area decrease of 10F, SDS-PAGE analysis of the SEC fractions confirmed the co-elution of 10F in the TNF-alpha peak upon prolonged incubation (Fig. 5C).

### Structural information on the ubiquitin variant 10F

**Figure 6:**
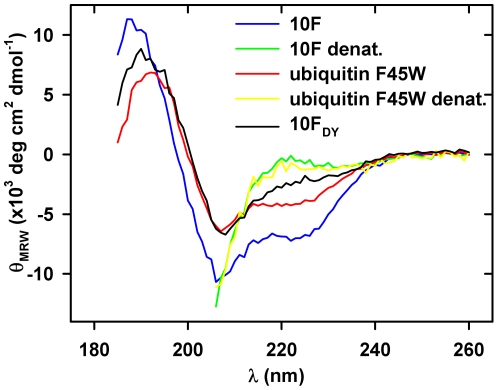
Circular dichroism analysis of ubiquitin variants. The far-UV CD spectra of the native proteins 10F (open circles), ubiquitin F45W (open triangles), and 10F_DY_ (open squares) were recorded in 10 mM KH_2_PO_4_ pH 7.4 at 20°C. For analysis of denatured proteins (closed symbols, 10F_DY_ not shown), samples were measured in 6 M guanidine hydrochloride. All spectra are buffer corrected.

Due to the substitution of 12% of the original sequence of the scaffold ubiquitin F45W in the selected variant 10F the overall structure of the protein might be affected. To investigate this, structural information on the selected binder was obtained. For secondary structure analysis of ubiquitin F45W and its two variants 10F and 10F_DY_ far-UV circular dichroism (CD) spectroscopy was used. All three proteins showed a negative band at 207±1 nm with the same intensity for ubiquitin F45W and 10F_DY_ but approximately 70% higher intensity for 10F (Fig. 6). A shoulder at 222 nm was observed for all proteins although less pronounced for 10F_DY_. Furthermore, a positive band was observed at 192 nm for ubiquitin F45W, 187 nm for 10F and 190 nm for 10F_DY_. After unfolding of ubiquitin F45W and 10F in 6 M guanidine hydrochloride, identical spectra were obtained for both proteins. The differences in the CD spectra of all three proteins suggest that especially the TNF-binding 10F may possess an altered structure compared to ubiquitin F45W. However, a detailed interpretation of these spectral differences is difficult because changes in the number of aromatic amino acids or their environment between the different proteins may significantly influence the far-UV CD spectra.

Far-UV CD spectroscopy was also employed to follow thermal un- and refolding of 10F. Measurements at 200 nm showed transition midpoints for unfolding at 75.9±0.2°C and for refolding at 72.4±0.2°C (Fig. S5). 10F_DY_ and ubiquitin F45W did not unfold completely under the experimental conditions. Far-UV CD spectra of 10F recorded before and after two cycles of heating to 90°C and cooling to 20°C showed no differences in shape or intensity (data not shown). Together, a reversible heat induced unfolding transition can be concluded. Probing the tertiary structure of 10F and 10F_DY_ by tryptophan fluorescence, thermal denaturation experiments resulted in T_m_ values of 69.0±0.1°C (10F) and 81.1±0.2°C (10F_DY_) (data not shown). Due to incomplete denaturation no T_m_ value was obtained for ubiquitin F45W.

**Figure 7:**
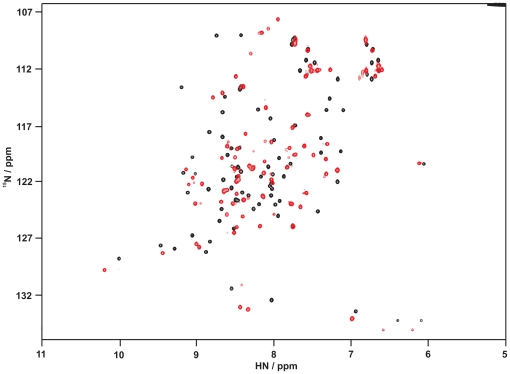
2D-NMR characterization of the scaffold ubiquitin F45W and the variant 10F. ^1^H-^15^N fHSQC spectra of the scaffold ubiquitin F45W (black, 900 µM) and its variant 10F (red, 400 µM) were acquired on an 800 MHz NMR spectrometer at 25°C. Both proteins were dissolved in PBS, 1 mM EDTA at pH 7.4. Superimposition of the spectra shows significant deviation of chemical shifts for almost all amino acids including the conserved ones. Note, that most residues between 130 ppm and 137 ppm are aliased along the ^15^N-dimension.

A more qualitative information about the overall protein fold for the scaffold protein ubiquitin F45W and the two variants 10F and 10F_DY_ was obtained from ^1^H-^15^N fHSQC spectra. ^15^N-labeled proteins were produced in auto-induction medium N-5052 [25] and purified using the identical procedure as for the unlabeled proteins. ^1^H-^15^N fHSQC spectra of all three proteins, each showing well dispersed cross peaks with narrow line shapes (Fig. 7, Fig. S2), indicate fully folded proteins with pronounced secondary and tertiary structure elements. A cross peak assignment for the scaffold ubiquitin F45W was performed on the basis of reported values for the ubiquitin wildtype [31]–[33]. Although the two variants 10F and 10F_DY_ showed well dispersed ^1^H-^15^N correlation spectra, their individual chemical shift positions differ clearly compared to the resonances for the scaffold protein. Due to this the peak assignment of the ubiquitin F45W spectrum could not be transferred to the spectrum of 10F. Consequently, structure determination of 10F in its soluted state as well as bound to TNF-alpha requires a completely new peak assignment. Even so, the spectral comparison suggests subtle differences of the tertiary structure between the scaffold protein and the two variants 10F and 10F_DY_.

## Discussion

To overcome the limitations of antibodies, which restrict their use for several biotechnological and pharmaceutical applications, artificial binding proteins based on alternative scaffolds were proven to be a valuable concept. Besides showing advantageous properties regarding architecture, stability and production [2], these alternative scaffolds offer the possibility to target epitopes that are not accessible for antibodies. The large size of the framework of antibodies and the concave shape of their paratope restrict the access to clefts and crypts of proteins, where often e.g. enzymatic active sites are located [34], while artificial binding proteins can bind such hidden epitopes [35]. In this study, an artificial binding protein based on the scaffold ubiquitin F45W was selected against TNF-alpha and its binding behavior towards the target protein was characterized. In order to create a new species of artificial binding molecules an already known binding site on the scaffold was modified to obtain new binding properties. Such a strategy proved to be successful for a range of previously presented scaffolds [5], [36]. In contrast to other artificial binding proteins utilizing loop or alpha-helical structures for the creation of binding surfaces, the technology applied here focuses particularly on the use of beta-sheets for this purpose.

According to these premises, six amino acid positions in the ubiquitin F45W were selected for randomization. From the resulting library the variant 10F, binding to the target protein TNF-alpha, was selected by ribosome display. Interestingly, in addition to the six expected substitutions (K6F, L8Y, R42A, I44E, H68P, V70F) two deletions (D58 & Y59) and one additional substitution (K29R) were found. The deletions were shown to be a critical factor for binding as demonstrated by the non-binding variant 10F_DY_ with the re-inserted amino acids. For the additional substitution K29R, we do not expect a target-specific interaction because of its position and conservative character. The selected variant 10F specifically binds its target protein TNF-alpha with a K_D_ of 0.18±0.05 µM, an affinity which is two to three orders of magnitude higher than most interactions of ubiquitin with ubiquitin-binding domains [15], [16].

### A new binding mode

The data from isothermal titration calorimetry showed an exothermic binding reaction and also indicated the binding of one 10F molecule to one TNF-alpha trimer, which constitutes the bioactive TNF species [37]. The 1∶3 stoichiometry was confirmed by analytical ultracentrifugation and NMR spectroscopy. Binding of more than one 10F molecule per TNF-alpha trimer was not observed, which excludes the binding to a single monomer of TNF-alpha. This contrasts the findings for the clinically approved anti-TNF-alpha antibody infliximab [38] and other monoclonal antibodies [39]. These bind both the trimer and the monomer species of TNF-alpha and thus show a 1∶1 stoichiometry. Also, binding proteins based on various alternative scaffolds, e.g. affibodies [9] and tetranectins [4], capable of recognizing the TNF-alpha trimer, show binding stoichiometries of 1∶1 per TNF-alpha monomer. Even TNF-receptors, recognizing exclusively the trimeric form, exhibit a 1∶1 stoichiometry as their epitope is presented three times by the trimer. This strongly indicates the recognition of a different, trimer-based epitope by the 10F molecule. According to our knowledge, such a binding mode to TNF-alpha has never been observed for protein-based binders before. A binding mode similar to small molecule-based inhibitors of TNF-alpha, which promote trimer dissociation [30], [40], could be excluded by fluorescence homoquenching experiments. However, a small-molecule inhibitor for the TNF family cytokine CD40 ligand seems to show an interaction mode, comparable to 10F [41]. The CD40 ligand inhibitor binds in a crypt between the subunits of the trimer. Maybe the low molecular mass of 10F with 9 kDa allows the artificial binding protein to access a similar crypt in TNF-alpha, which is inaccessible for antibodies due to their molecular mass of more than 120 kDa.

In NMR titration and SEC experiments an influence of the detergent Tween-20 on the interaction between the selected binder and TNF-alpha was discovered. A similar observation was reported for DARPins [8]. The investigation of this detergent effect showed that the ubiquitin variant 10F binds TNF-alpha regardless of the presence or absence of Tween-20, but the detergent accelerates the binding kinetics. Interestingly, the time range necessary to observe complex formation of the ubiquitin variant and TNF-alpha under detergent-free conditions coincides quite well with the values reported for the TNF-alpha trimer dissociation half-time in absence of detergents ranging from 4 to 20 h [40], [42]. A conformational transition is known to precede the dissociation of the trimeric molecule [40]. Such local conformational changes in TNF-alpha were observed at low Tween-20 concentrations of up to 0.1% (v/v) using ^1^H-NMR spectroscopy (data not shown) and are probably necessary for binding of 10F. From this, we suggest a binding model where Tween-20 mediates the association event by stabilizing a particular conformational state of the TNF-alpha trimer, which is bound by 10F. Remarkably, this model is not in contradiction to the successful competition of the interaction with etanercept. The TNF-receptor 2 may bind a different conformation, which disallows binding of 10F.

Interestingly, Tween-20 sensitivity is not totally unknown for antibodies against TNF-alpha, the reported example shows the suppression of binding in the presence of Tween-20 [43]. However, for a variety of successful selections against TNF-alpha with antibodies and alternative scaffolds, so far no detergent sensitivity of the binders was reported [4], [9]. In these cases in contrast to the present study, Tween-20 was substituted by other detergents at least during parts of the selection procedure. Whether this avoids the selection of Tween-20-sensitive binding molecules remains unclear, as the here presented ubiquitin variant 10F is capable of interacting with TNF-alpha in the absence of Tween-20. Hence, a selection without Tween-20 or other detergents, respectively, may result in binding of the same epitope.

### The selected ubiquitin variant is structured and stable

The number of substitutions and the deletions in the ubiquitin-based scaffold that yielded the TNF-binding ubiquitin variant were supposed to influence the structure and stability compared to ubiquitin F45W. Although such a number of substitutions – 12% of the amino acid sequence – is likely to destroy the structure of a protein, this particular ubiquitin variant appeared in its structural characterization by CD and NMR spectroscopy to have typical properties of a natively folded protein. However, differences in the spectroscopic character were obvious. Differences in the far-UV CD spectrum at around 224 nm in 10F_DY_ compared to the scaffold ubiquitin F45W may arise from the additional tyrosine residue Y8, giving a positive signal in the CD spectrum. Such distinctive features are well described for proteins displaying a low content of helical structures and may also explain the characteristic spectrum of 10F [44], [45]. In the NMR spectra, significant differences of the individual chemical shifts were observed for both variants 10F and 10F_DY_ compared to the scaffold ubiquitin F45W. From these data alone it is difficult to predict the extent of structural rearrangements by the substitutions. Such conclusions require a highly resolved structure obtained by NMR spectroscopy or X-ray crystallography, which is currently in progress.

One of the advantageous properties of ubiquitin making it an interesting scaffold is its high thermal stability of more than 90°C [46]. In comparison, the fully reversible temperature induced unfolding of the binder 10F followed by far-UV circular dichroism and tryptophan fluorescence (data not shown) showed transition midpoints at around 76°C and 69°C, respectively. The deletion of D58 and Y59 shows a large contribution to thermal stability as 10F_DY_ shows a 12 K increased T_m_ compared to 10F. Even with a significantly decreased transition temperature compared to the ubiquitin wildtype, 10F still fairly competes with other scaffold-derived proteins, for example anticalins, DARPins, and gammaB-crystallin-based binders, regarding thermal stability [3], [6], [11].

### Conclusions

In summary, we showed a second example of creating highly stable artificial binding proteins with a binding site on beta-sheet structures, in addition to the gammaB-crystallin-based binders [11]. From a library based on the scaffold ubiquitin we were able to select a variant binding specifically to the TNF-alpha trimer. The detergent Tween-20 mediates the interaction, but is not required for binding. The particular epitope targeted by this new binding mode appears to differ from those reported for TNF-neutralizing monoclonal antibodies [47]. This unique binding behavior underlines the potential of alternative scaffolds to obtain artificial binding proteins targeting epitopes which might be hardly accessible to antibodies. For the future, this offers the opportunity to select an appropriate scaffold for distinct epitopes and targets.

## Supporting Information

Figure S1 **Binding of different ubiquitin variants to TNF-alpha analyzed by ELISA.** Binding of the ubiquitin variants 10F and 10F_DY_ (latter one derived by re-insertion of two deleted amino acids), and the scaffold ubiquitin F45W to immobilized TNF-alpha (150 ng per well) are depicted.
(TIF)

Figure S2 **^1^H-^15^N fHSQC spectra of ubiquitin F45W and its variants 10F and 10F_DY_.** Spectra were recorded with ^15^N-labeled proteins dissolved in PBS, 1 mM EDTA pH 7.4. (A) Free and TNF-alpha-bound state of the ubiquitin variant 10F. Measurements were performed with 160 µM 10F in presence of 0.05% (v/v) Tween-20. The spectra of the free (black) and the bound (4-fold excess of TNF-alpha trimer) state (red) were superimposed. (B) Comparison of ubiquitin F45W and its variant 10F_DY_. Protein concentrations were fixed to 900 µM (ubiquitin F45W, black) and 600 µM (10F_DY_, red). (C) Comparison of ubiquitin variants 10F_DY_ and 10F. Spectra were recorded with 600 µM 10F_DY_ (black) and 400 µM 10F (red). Note, that in (B) and (C) most residues between 130 ppm and 137 ppm are aliased along the ^15^N-dimension and that in (A), the ^15^N-spectral width was further reduced.
(TIF)

Figure S3 **Determination of the molecular mass of the TNF-alpha-10F complex.** Molecular mass of fluorescein-labeled 10F (open circles) and TNF-alpha-10F complex (closed circles) was determined by analytical ultracentrifugation. Upper panel: absorbance data (490 nm) were fitted to a single species model. The fits yielded calculated molecular masses of 11±1 kDa for 10F and 65±5 kDa for the complex (theoretical molecular mass of 10F: 9.9 kDa and complex: 62.0 kDa). Lower panel: deviation of the fits to the experimental data.
(TIF)

Figure S4 **Inhibition of TNF-alpha subunit dissociation analyzed by fluorescence homoquenching assay.** The increase in fluorescence dequenching of 100 nM fluorescein-labeled TNF-alpha incubated with a 200-fold excess of unlabeled TNF-alpha was used to follow subunit dissociation of TNF-alpha trimers. After 38 min (dashed line) 5 µl 10F (595 µM), or one of the controls PBS, 10F_DY_ (600 µM) and etanercept (160 µM) were added to a total volume of 155 µl.
(TIF)

Figure S5 **Thermal denaturation of the TNF-alpha binding ubiquitin variant 10F.** By following the circular dichroism signal at 200 nm, we measured the thermal unfolding and refolding of the ubiquitin variant 10F in 10 mM KH_2_PO_4_ pH 7.0. T_m_ values of 75.9±0.2°C for unfolding and 72.4±0.2°C for refolding were determined. Ubiquitin F45W and 10F_DY_ were not denatured completely under the applied conditions (data not shown). In corresponding fluorescence measurements in PBS, 1 mM EDTA at pH 7.4 – probing the tertiary structure of 10F and 10F_DY_ by intrinsic tryptophan fluorescence – unfolding T_m_ values of 69.0±0.1°C (10F) and 81.1±0.2°C (10F_DY_) were found (data not shown). The thermal stability of ubiquitin F45W allowed no complete denaturation under the used conditions (T_m_ above 90°C).
(TIF)

Methods S1 **Additional methods.**
(PDF)
