# Supplementary figures and images for: New Binding Mode to TNF-Alpha Revealed by Ubiquitin-Based Artificial Binding Protein
Source: PLoS One. 2012 Feb 20;7(2):e31298. doi: 10.1371/journal.pone.0031298 (PMC3282696; doi:10.1371/journal.pone.0031298)

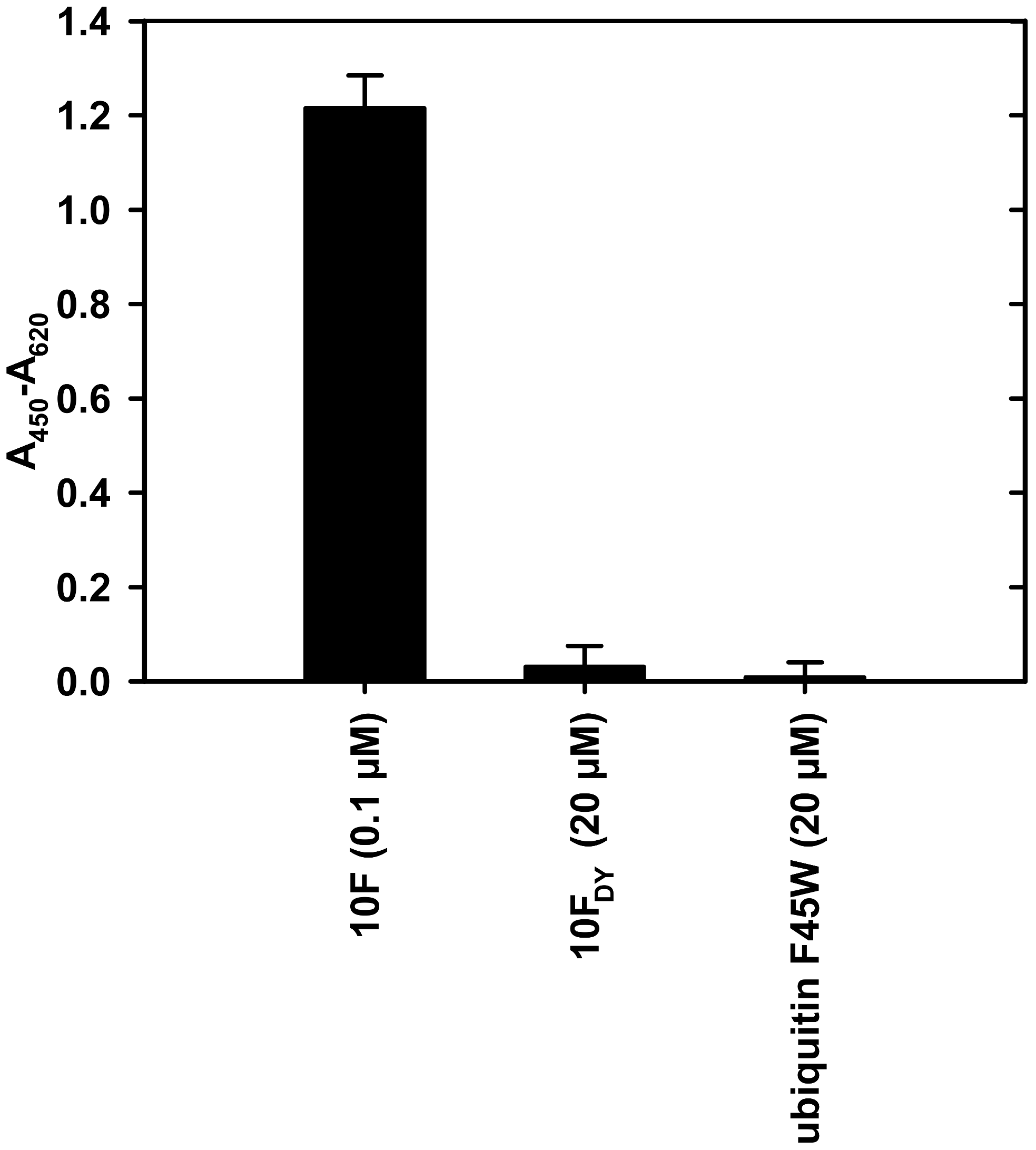

Supplement: Figure S1 — Binding of different ubiquitin variants to TNF-alpha analyzed by ELISA. Binding of the ubiquitin variants 10F and 10FDY (latter one derived by re-insertion of two deleted amino acids), and the scaffold ubiquitin F45W to immobilized TNF-alpha (150 ng per well) are depicted. (TIF) [file pone.0031298.s001.tif]

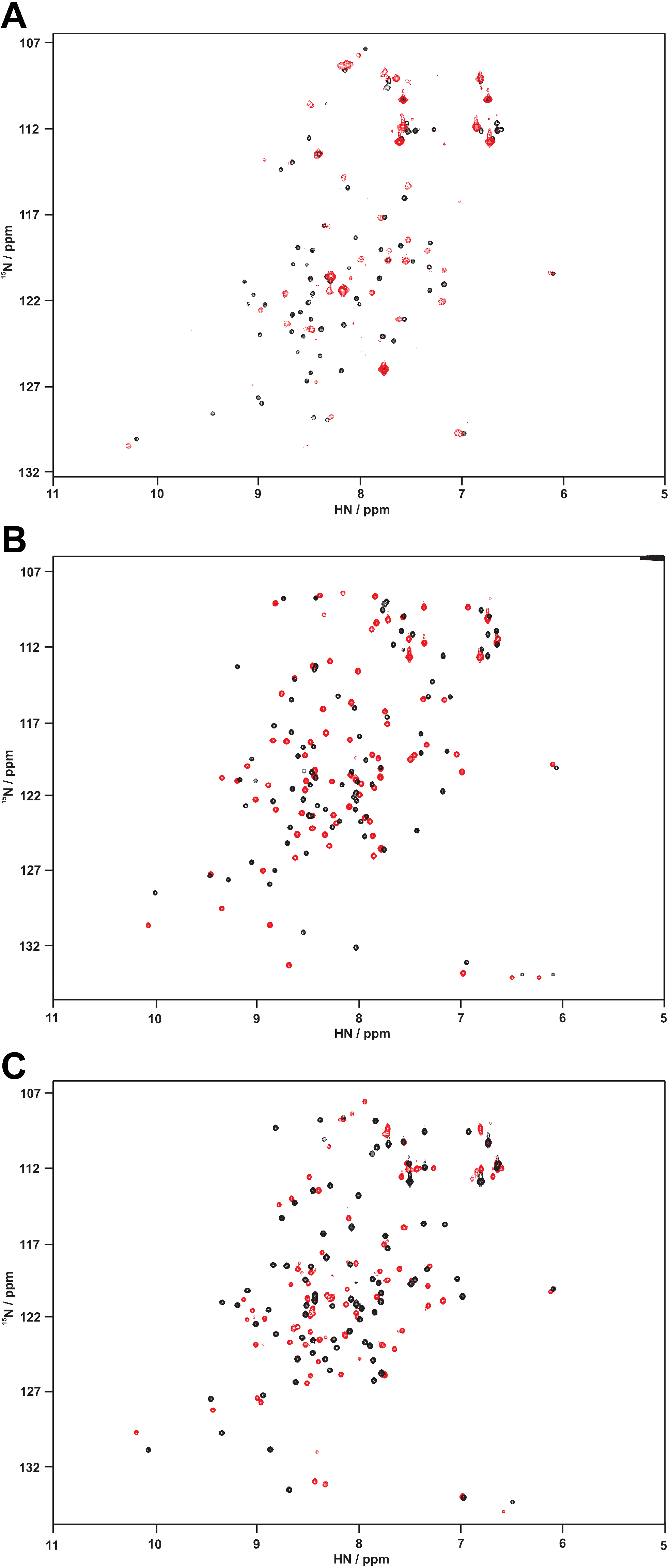

Supplement: Figure S2 — 1H-15N fHSQC spectra of ubiquitin F45W and its variants 10F and 10FDY. Spectra were recorded with 15N-labeled proteins dissolved in PBS, 1 mM EDTA pH 7.4. (A) Free and TNF-alpha-bound state of the ubiquitin variant 10F. Measurements were performed with 160 µM 10F in presence of 0.05% (v/v) Tween-20. The spectra of the free (black) and the bound (4-fold excess of TNF-alpha trimer) state (red) were superimposed. (B) Comparison of ubiquitin F45W and its variant 10FDY. Protein concentrations were fixed to 900 µM (ubiquitin F45W, black) and 600 µM (10FDY, red). (C) Comparison of ubiquitin variants 10FDY and 10F. Spectra were recorded with 600 µM 10FDY (black) and 400 µM 10F (red). Note, that in (B) and (C) most residues between 130 ppm and 137 ppm are aliased along the 15N-dimension and that in (A), the 15N-spectral width was further reduced. (TIF) [file pone.0031298.s002.tif]

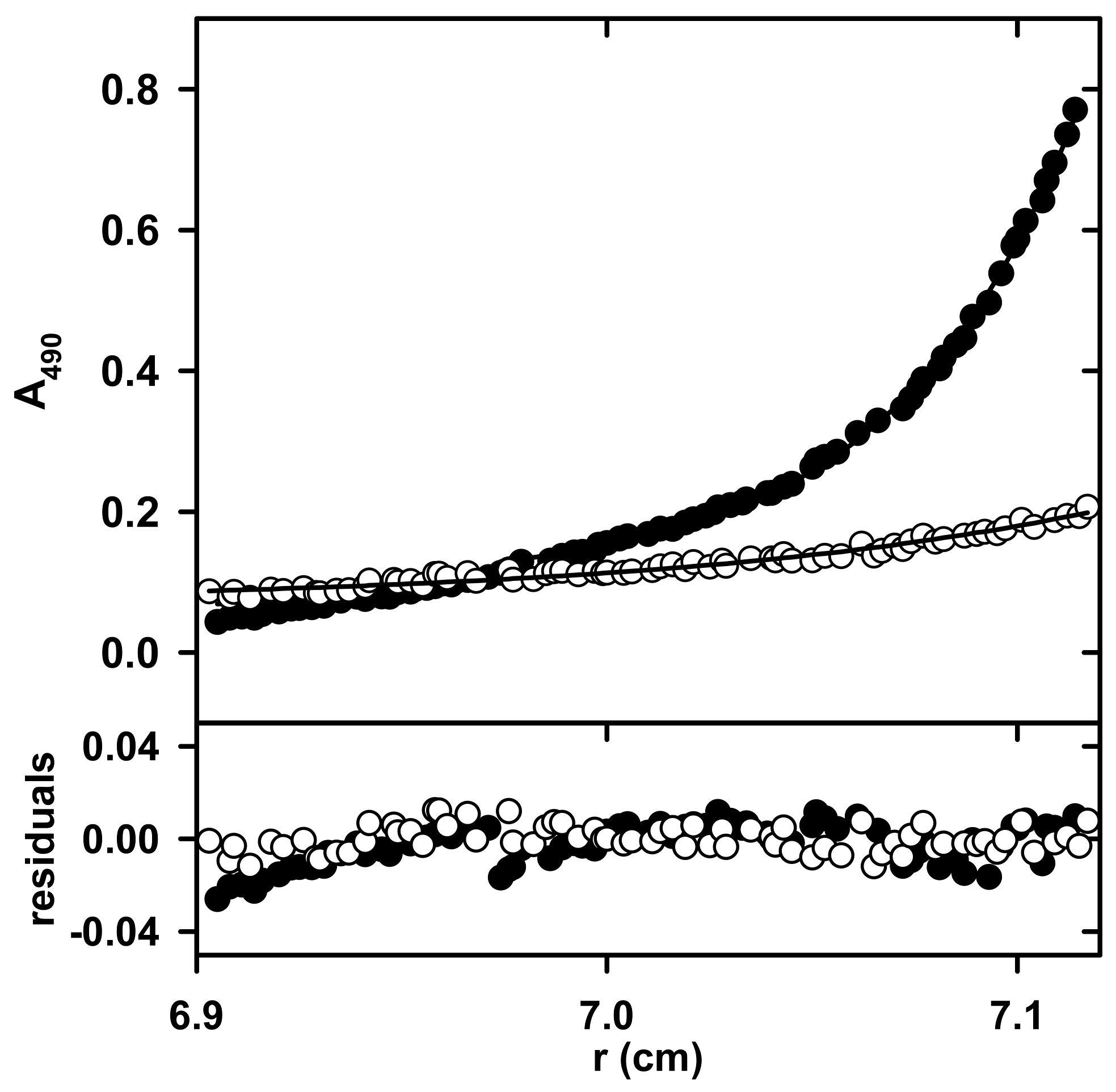

Supplement: Figure S3 — Determination of the molecular mass of the TNF-alpha-10F complex. Molecular mass of fluorescein-labeled 10F (open circles) and TNF-alpha-10F complex (closed circles) was determined by analytical ultracentrifugation. Upper panel: absorbance data (490 nm) were fitted to a single species model. The fits yielded calculated molecular masses of 11±1 kDa for 10F and 65±5 kDa for the complex (theoretical molecular mass of 10F: 9.9 kDa and complex: 62.0 kDa). Lower panel: deviation of the fits to the experimental data. (TIF) [file pone.0031298.s003.tif]

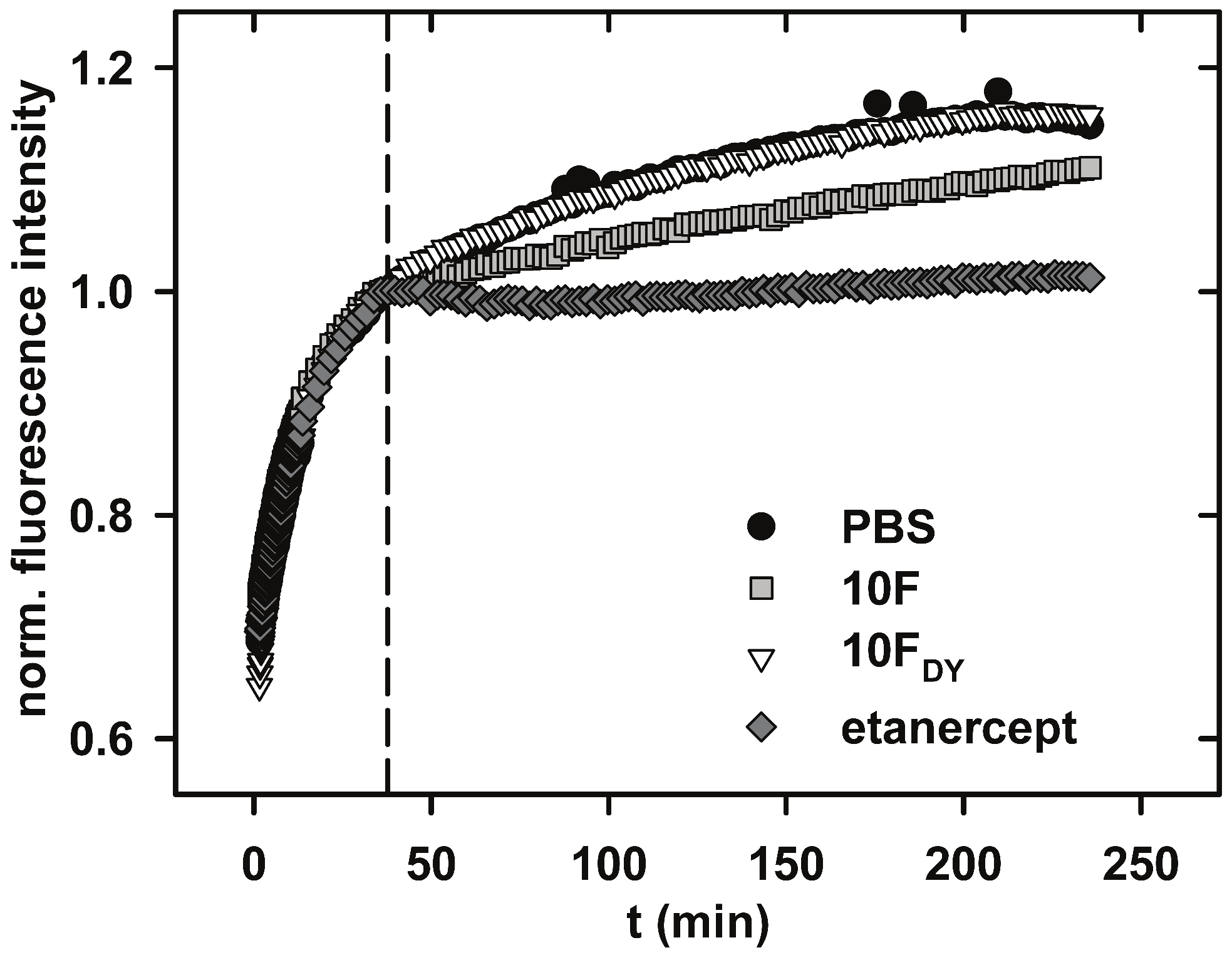

Supplement: Figure S4 — Inhibition of TNF-alpha subunit dissociation analyzed by fluorescence homoquenching assay. The increase in fluorescence dequenching of 100 nM fluorescein-labeled TNF-alpha incubated with a 200-fold excess of unlabeled TNF-alpha was used to follow subunit dissociation of TNF-alpha trimers. After 38 min (dashed line) 5 µl 10F (595 µM), or one of the controls PBS, 10FDY (600 µM) and etanercept (160 µM) were added to a total volume of 155 µl. (TIF) [file pone.0031298.s004.tif]

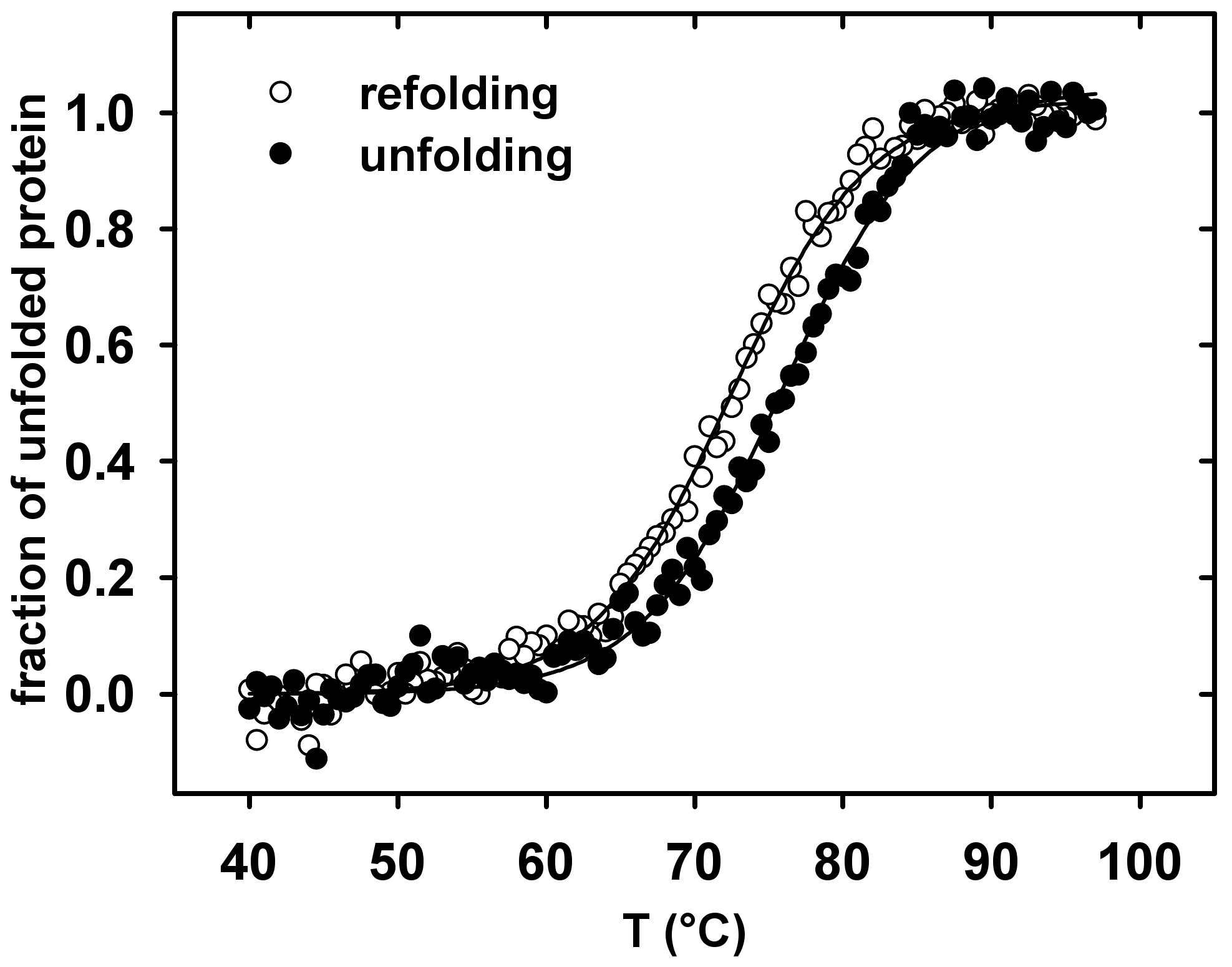

Supplement: Figure S5 — Thermal denaturation of the TNF-alpha binding ubiquitin variant 10F. By following the circular dichroism signal at 200 nm, we measured the thermal unfolding and refolding of the ubiquitin variant 10F in 10 mM KH2PO4 pH 7.0. Tm values of 75.9±0.2°C for unfolding and 72.4±0.2°C for refolding were determined. Ubiquitin F45W and 10FDY were not denatured completely under the applied conditions (data not shown). In corresponding fluorescence measurements in PBS, 1 mM EDTA at pH 7.4 – probing the tertiary structure of 10F and 10FDY by intrinsic tryptophan fluorescence – unfolding Tm values of 69.0±0.1°C (10F) and 81.1±0.2°C (10FDY) were found (data not shown). The thermal stability of ubiquitin F45W allowed no complete denaturation under the used conditions (Tm above 90°C). (TIF) [file pone.0031298.s005.tif]
