# Supplementary material for: New Binding Mode to TNF-Alpha Revealed by Ubiquitin-Based Artificial Binding Protein
Source: PLoS One. 2012 Feb 20;7(2):e31298. doi: 10.1371/journal.pone.0031298 (PMC3282696; doi:10.1371/journal.pone.0031298)
Supplement: Methods S1 — Additional methods. (PDF) [file pone.0031298.s006.pdf]

# New binding mode to TNF-alpha revealed by ubiquitin-based artificial binding protein

## Supplementary methods

### Chemical modification of proteins with fluorescein

5-(and 6-)carboxyfluorescein-succinimidyl ester (Pierce Biotechnology, Rockford, IL) or 6-(fluorescein-5-carboxamido)-hexanoic acid-succinimidyl ester (Invitrogen, Carlsbad, CA) was used for fluorescent labeling of the proteins. To obtain preferentially N-terminally labeled proteins, reactions were performed at pH 6.5. Further reaction conditions were kept according to manufacturer's instructions. Modification was confirmed by SDS-PAGE and mass spectrometry analysis.

### Analytical ultra-centrifugation

Sedimentation equilibrium measurements were performed for 70 h in an analytical ultracentrifuge Optima XL-A (Beckman Instruments, Inc., Fullerton, CA). Double sector cells were used at 9,000 rpm and 20 °C in an An60Ti rotor. Analyses were carried out at a protein concentration of 0.04 mg $ml^{-1}$ (4 µM) for fluorescein-labeled 10F in PBS, 1 mM EDTA pH 7.4, supplemented with 0.05 % (v/v) Tween-20 in absence or presence of 54 µM TNF-alpha utilizing fluorescein absorbance at 490 nm. The data obtained were analyzed with a program provided by Beckman Instruments. For calculations, a partial specific volume of 0.73 ml $mg^{-1}$ was assumed.

### TNF-alpha subunit dissociation analysis by fluorescence homoquenching assay

Microtiter plate wells (Greiner Standard 96F Black Non-binding) were filled with 30 µl of 500 nM fluorescein-labeled TNF-alpha solution. After addition of 120 µl unlabeled TNF-alpha, resulting in a 200-fold excess of unlabeled TNF-alpha (final concentration: 20 µM), a time course of fluorescence emission intensity was measured (excitation filter: 485 nm, emission filter: 535 nm). After 38 min of incubation, 5 µl of PBS or protein solutions of 595 µM 10F, 600 µM $10F_{DY}$ or 160 µM etanercept were added to the wells, plates were shaken for 3 s and fluorescence measurements were continued. Experiments were performed in PBS, 1 mM EDTA pH 7.4, supplemented with

0.05 % (v/v) Tween-20. To prevent evaporation, plates were lidded. Data were normalized to the fluorescence intensity at 38 min.

**Thermal denaturation analysis by far-UV circular dichroism**

Thermal denaturation experiments were conducted in a temperature range of 15-97 °C using a slope of 1 K $min^{-1}$. Measurements were performed on a Jasco J-810 spectropolarimeter (Jasco Inc., Easton, MD) with a 1 mm quartz cuvette. The used protein concentration was 11.87 µM and the detection wavelength was set to 200 nm. Data were normalized according to Pace and Scholtz [1].

**Supplementary reference**

1. Pace CN, Scholtz JM (1997) Measuring the conformational stability of a protein. In: Creighton TE, editor. Protein Structure: A practical approach. 2nd ed. Oxford, UK: Oxford University Press. pp. 299-321
